# Supplementary material for: Attentional Functioning in Healthy Older Adults and aMCI Patients: Results from the Attention Network Test with a Focus on Sex Differences
Source: Brain Sci. 2025 Jul 19;15(7):770. doi: 10.3390/brainsci15070770 (PMC12293916; doi:10.3390/brainsci15070770)
Supplement: Supplementary file 1 [file brainsci-15-00770-s001.zip › brainsci-3726821-supplementary.pdf]

Supplementary materials

S1: Descriptive statistics of mean RTs from the ANT.

Table S1.1. Descriptive statistics of mean RTs from the ANT. Mean (and SD) of average RTs, stratified by Health Condition (MCI vs. HOCs), Sex (female vs male) and Health Condition * Sex. Means are reported for all trials, independently from flankers and cues types.

	Total RTs
**Health Condition**	
MCI	892.97 (148.11)
HOCs	732.76 (108.01)
**Sex**	
Females	857.96 (160.49)
Males	756.49 (119.83)
**Health Condition*Sex**	
MCI_Females	971.47 (127.2)
MCI_Males	794.84 (108.91)
HOCs_Females	744.45 (97.78)
HOCs_Males	718.15 (118.48)

Table S1.2. Descriptive statistics of mean RTs from the ANT. Mean (and SD) of average RTs, stratified by Flanker Congruency (congruent, incongruent) on the left, and Cue Type (no cue, double cue, central cue, spatial cue) on the right. Means are reported for all participants, regardless of Health Condition and Sex.

Flanker Congruency		Cue type			
**Congruent**	**Incongruent**	**No cue**	**Double cue**	**Central cue**	**Spatial cue**
795.47 (136.1)	906.69 (140.68)	838.97 (142.3)	806.01 (155.09)	812.63 (156.81)	793.94 (153.01)

S2: Full results of the Generalized Linear Mixed Model (GLMM) with custom family, examining the Health Condition * Sex interaction effects on mean raw RTs.

Table S2.1: Fixed effect omnibus tests of the GLMM conducted on raw RTs. A GLMM was employed to predict the response time in milliseconds based on Health Condition (MCI and HOCs), Sex (females and males), Flanker Congruency (congruent, incongruent and neutral trials), Cue Type (no cue, double cue, central cue and spatial cue) and number of errors, following the formula: RT ~ 1 + Health Condition + Sex + Cue Type + Flanker Congruency + Errors + Health Condition * Sex + Health Condition * Cue Type + Sex * Cue Type + Health Condition * Flanker Congruency + Sex * Flanker Congruency + Cue * Flanker Congruency + Health Condition * Sex * Cue Type + Health Condition * Sex * Flanker Congruency + Health Condition * Cue Type * Flanker Congruency + Sex * Cue Type * Flanker Congruency + Health Condition * Sex * Cue Type * Flanker Congruency + (1 | Participant ID). Error numeric covariate was mean-centered. A p-value (*p*) < 0.05 was considered statistically significant and is highlighted in bold. The model incorporated Participant ID as a random intercept, represented by the formula: ~1 | Participant ID. df: degrees of freedom.

**Predictor**	**X^2**	**df**	***p***
Health Condition	117.42	1.00	**<.001**
Sex	53.39	1.00	**<.001**
Cue Type	59.90	3.00	**<.001**
Flanker Congruency	903.28	2.00	**<.001**
Number of errors	0.71	1.00	0.398
Health Condition * Sex	36.13	1.00	**<.001**
Health Condition * Cue Type	3.64	3.00	0.303
Sex * Cue Type	5.48	3.00	0.140
Health Condition * Flanker Congruency	2.91	2.00	0.233
Sex * Flanker Congruency	2.73	2.00	0.256
Cue Type * Flanker Congruency	30.56	6.00	**<.001**
Health Condition * Sex * Cue Type	1.20	3.00	0.754
Health Condition * Sex * Flanker Congruency	1.44	2.00	0.487
Health Condition * Cue Type * Flanker Congruency	7.42	6.00	0.284
Sex * Cue Type * Flanker Congruency	4.24	6.00	0.644
Health Condition * Sex * Cue Type * Flanker Congruency	1.94	6.00	0.925

Table S2.2: Fixed effects parameter estimates of predictors and interactions showing a significant effect on raw RTs. A p-value (p) < 0.05 was considered statistically significant. CI: Confidence Interval; SE: Standard Error; MCI: patients with Mild Cognitive Impairment; HOCs: Healthy Older Controls. M: males; F: females; 1: no cue; 2: double cue; 3: central cue; 4: spatial cue; Congruency: Flanker Congruency; -1: incongruent trials; 1: congruent trials; 0: neutral trials.

				95% CI			
**Predictor**	**Effect**	**Estimate**	**SE**	**Lower**	**Upper**	**z**	***p***
(Intercept)	(Intercept)	816.82 ms	6.78	803.53	830.10	120.51	<.001
Health Condition	MCI – HOCs	153.93 ms	14.21	126.09	181.78	10.84	<.001
Sex	M – F	-99.38 ms	13.60	-126.03	-72.72	-7.31	<.001
Cue Type	2 – 1	-28.00 ms	6.34	-40.42	-15.58	-4.42	<.001
	3 – 1	-34.08 ms	6.32	-46.47	-21.69	-5.39	<.001
	4 – 1	-47.43 ms	6.27	-59.72	-35.13	-7.56	<.001
Congruency	0 – (-1)	-169.51 ms	5.64	-180.57	-158.44	-30.03	<.001
	1 – (-1)	-111.75 ms	5.81	-123.13	-100.37	-19.25	<.001
Health Condition * Sex	MCI – HOCs * M – F	-163.77 ms	27.25	-217.18	-110.37	-6.01	<.001
Sex * Cue Type	M – F * 2 – 1	-24.81 ms	12.67	-49.64	0.03	-1.96	0.050
Sex * Cue Type	M – F * 4 – 1	-26.55 ms	12.53	-51.12	-1.98	-2.12	0.034
Cue Type * Congruency	2 – 1 * 0 – (-1)	-55.56 ms	15.69	-86.31	-24.81	-3.54	<.001
Cue Type * Congruency	3 – 1 * 0 – (-1)	-62.30 ms	15.71	-93.09	-31.52	-3.97	<.001
Cue Type * Congruency	3 – 1 * 1 – (-1)	-58.00 ms	16.15	-89.67	-26.34	-3.59	<.001

Table S2.3: Post Hoc comparisons of relevant predictors exerting a significant effect on raw RTs. (A) Post Hoc comparisons of Sex * Health Condition. **(B)** Post Hoc Comparisons of Cue Type. **(C)** Post Hoc Comparisons of Flanker Congruency. A p-value (*p*) < 0.05 Bonferroni corrected was considered statistically significant and highlighted in bold. Δ: estimated marginal means difference; SE: Standard Error; HC: Health Condition; MCI: patients with Mild Cognitive Impairment; HOCs: Healthy Older Controls. M: males; F: females. 1: no cue; 2: double cue; 3: central cue; 4: spatial cue; -1: incongruent trials; 1: congruent trials; 0: neutral trials.

(A)

Comparison							
**Sex**	**HC**	**Sex**	**HC**	**Δ**	**SE**	**z**	***p bonf***
F	HOCs	F	MCI	-235.8 ms	19.1	-12.37	**< .001**
F	HOCs	M	HOCs	17.5 ms	19.0	0.92	1.000
F	HOCs	M	MCI	-54.6 ms	19.3	-2.82	**0.029**
F	MCI	M	MCI	181.3 ms	19.5	9.29	**< .001**
M	HOCs	F	MCI	-253.3 ms	20.0	-12.67	**< .001**
M	HOCs	M	MCI	-72.0 ms	20.3	-3.55	**0.002**

(B)

Comparison					
**Cue Type**	**Cue Type**	**Δ**	**SE**	**z**	***p bonf***
1	2	28.00 ms	6.34	4.42	**< .001**
1	3	34.08 ms	6.32	5.39	**< .001**
1	4	47.43 ms	6.27	7.56	**< .001**
2	3	6.07 ms	6.20	0.98	1.000
2	4	19.42 ms	6.15	3.16	**0.010**
3	4	13.35 ms	6.12	2.18	0.176

(C)

Comparison					
**Congruency**	**Congruency**	**Δ**	**SE**	**z**	***p bonf***
-1	0	169.5 ms	5.64	30.0	**< .001**
-1	1	111.7 ms	5.81	19.3	**< .001**
0	1	-57.8 ms	5.06	-11.4	**< .001**

S3: Full results of the Mixed Model fit by REML, examining the Health Condition * Sex interaction effects on z-scored RTs.

Table S3.1: Fixed effect omnibus tests of Mixed Model conducted on z-scored RTs. A Mixed Model was employed to predict the corrected response time in milliseconds based on Health Condition (MCI and HOCs), Sex (females and males), Flanker Congruency (congruent, incongruent and neutral trials), Cue Type (no cue, double cue, central cue and spatial cue) and number of errors, following the formula: z-scored RTs~ 1 + Health Condition + Sex + Cue Type + Flanker Congruency + Errors + Health Condition * Sex + Health Condition * Cue Type + Sex * Cue Type + Health Condition * Flanker Congruency + Sex * Flanker Congruency + Cue * Flanker Congruency + Health Condition * Sex * Cue Type + Health Condition * Sex * Flanker Congruency + Health Condition * Cue Type * Flanker Congruency + Sex * Cue Type * Flanker Congruency + Health Condition * Sex * Cue Type * Flanker Congruency + (1 | Participant ID). Error numeric covariate was mean-centered. A p-value (*p*) < 0.05 was considered statistically significant and is highlighted in bold. The model incorporated Participant ID as a random intercept, represented by the formula: ~1 | Participant ID. Satterthwaite method for degrees of freedom (df) was employed. Num df: numerator df; Den df: denominator df.

Predictor	F	Num df	Den df	*p*
Health Condition	4.35	1	27.6	**0.046**
Sex	5.17	1	25.0	**0.032**
Cue Type	28.37	3	344.2	**< .001**
Flanker Congruency	647.42	2	351.9	**< .001**
Number of errors	104.34	1	47.0	**< .001**
Health Condition * Sex	4.95	1	25.1	**0.035**
Health Condition * Cue Type	1.40	3	344.5	0.244
Sex * Cue Type	2.41	3	344.1	0.067
Health Condition * Flanker Congruency	0.57	2	348.0	0.565
Sex * Flanker Congruency	4.14	2	347.6	**0.017**
Cue Type * Flanker Congruency	7.36	6	344.2	**< .001**
Health Condition * Sex * Cue Type	0.37	3	344.0	0.778
Health Condition * Sex * Flanker Congruency	0.18	2	348.6	0.835
Health Condition * Cue Type * Flanker Congruency	1.22	6	344.5	0.296
Sex * Cue Type * Flanker Congruency	1.31	6	343.8	0.254
Health Condition * Sex * Cue Type * Flanker Congruency	0.55	6	344.1	0.774

Table S3.2: Fixed effects parameter estimates of predictors and interactions showing a significant effect on z-scored RTs. A p-value (p) < 0.05 was considered statistically significant. CI: Confidence Interval; SE: Standard Error; df: degrees of freedom; HC: Health Condition; MCI: patients with Mild Cognitive Impairment; HOCs: Healthy Older Controls. M: males; F: females; Cue: Cue Type; 1: no cue; 2: double cue; 3: central cue; 4: spatial cue; Congruency: Flanker Congruency; -1: incongruent trials; 1: congruent trials; 0: neutral trials.

				95% CI				
**Predictor**	**Effect**	**Estimate**	**SE**	**Lower**	**Upper**	**df**	**t**	***p***
HC	MCI – HOCs	-0.11 ms	0.05	-0.22	-0.01	27.6	-2.08	0.046
Sex	M – F	0.11 ms	0.05	0.06	0.21	25.0	2.27	0.032
Cue	2 – 1	-0.34 ms	0.06	-0.47	-0.22	344.2	-5.34	< .001
	3 – 1	-0.41 ms	0.06	-0.53	-0.28	344.6	-6.36	< .001
	4 – 1	-0.57 ms	0.06	-0.70	-0.45	344.8	-8.97	< .001
Congruency	0 – (-1)	-1.98 ms	0.06	-2.09	-1.87	357.2	-35.35	< .001
	1 – (-1)	-1.34 ms	0.06	-1.45	-1.23	356.3	-23.86	< .001
Number of errors	Errors	-0.11 ms	0.01	-0.13	-0.07	47.0	-10.21	< .001
HC * Sex	MCI – HOCs * M – F	0.23 ms	0.10	0.03	0.41	25.1	2.22	0.035
Sex * Cue	M – F * 2 – 1	-0.28 ms	0.13	-0.53	-0.03	344.0	-2.18	0.030
	M – F * 4 – 1	-0.31 ms	0.13	-0.56	0.06	344.6	-24.33	0.016
Sex * Congruency	M – F * 0 – (-1)	-0.29 ms	0.11	-0.51	-0.08	348.5	-2.64	0.009
	M – F * 1 – (-1)	-0.26 ms	0.11	-0.48	-0.04	350.5	-2.32	0.021
Cue * Congruency	2 – 1 * 0 – (-1)	-0.70 ms	0.16	-1.00	-0.39	345.1	-4.43	< .001
	3 – 1 * 0 – (-1)	0.79 ms	0.16	-1.10	-0.48	344.8	-5.05	< .001
	2 – 1 * 1 – (-1)	-0.43 ms	0.16	-0.74	0.12	344.7	-2.75	0.006
	3 – 1 * 1 – (-1)	-0.70 ms	0.16	-1.01	0.40	344.0	-4.49	< .001
HC * Cue * Congruency	MCI – HOCs * 2 – 1 * 1 – (-1)	0.64 ms	0.31	0.02	1.25	343.8	2.04	0.042
Sex * Cue * Congruency	M – F * 3 – 1 * 0 – (-1)	-0.76 ms	0.31	-1.37	-0.14	343.8	-2.40	0.016
	M – F * 3 – 1 * 1 – (-1)	-0.75 ms	0.31	-1.37	- 0.14	343.8	-2.40	0.017

Table S3.3: Post Hoc comparisons of relevant predictors exerting a significant effect on z-scored RTs. (A) Post Hoc comparisons of Health Condition * Sex. **(B)** Post Hoc Comparisons of Cue Type. **(C)** Post Hoc Comparisons of Flanker Congruency. **(D)** Post Hoc comparisons of Sex * Flanker Congruency. A p-value (*p*) < 0.05 Bonferroni corrected was considered statistically significant and highlighted in bold. Δ: estimated marginal means difference; SE: Standard Error; df: degrees of freedom; HC: Health Condition; MCI: patients with Mild Cognitive Impairment; HOCs: Healthy Older Controls. M: males; F: females. 1: no cue; 2: double cue; 3: central cue; 4: spatial cue; -1: incongruent trials; 1: congruent trials; 0: neutral trials.

(A)

**Comparison**								
**HC**	**Sex**	**HC**	**Sex**	**Δ**	**SE**	**t**	**df**	***p bonf***
HOCs	F	HOCs	M	-0.001 ms	0.07	-0.03	31.1	1.000
HOCs	F	MCI	F	0.22 ms	0.07	3.08	34.5	**0.024**
HOCs	F	MCI	M	0.001 ms	0.07	0.02	32.1	1.000
HOCs	M	MCI	M	0.003 ms	0.07	0.04	31.9	1.000
MCI	F	HOCs	M	-0.22 ms	0.08	-2.98	33.9	**0.032**
HOCs	F	MCI	M	-0.22 ms	0.07	-3.15	31.7	**0.021**

(B)

**Comparison**						
**Cue Type**	**Cue Type**	**Δ**	**SE**	**t**	**df**	***p bonf***
1	2	0.34 ms	0.06	5.34	352	**< .001**
1	3	0.41 ms	0.06	6.36	352	**< .001**
1	4	0.57 ms	0.06	8.97	352	**< .001**
2	3	0.07 ms	0.06	1.02	352	1.000
2	4	0.23 ms	0.06	3.63	352	**0.002**
3	4	0.17 ms	0.06	2.61	352	0.057

(C)

**Comparison**						
**Congruency**	**Congruency**	**Δ**	**SE**	**t**	**df**	***p bonf***
-1	0	1.98 ms	0.06	35.3	362	**< .001**
-1	1	1.34 ms	0.06	23.9	362	**< .001**
0	1	-0.65 ms	0.06	-11.6	352	**< .001**

(D)

Comparison								
**Sex**	**Congruency**	**Sex**	**Congruency**	**Δ**	**SE**	**t**	**df**	***p bonf***
F	-1	F	0	1.84 ms	0.08	24.42	366	**< .001**
F	-1	F	1	1.21 ms	0.08	16.05	367	**< .001**
F	-1	M	-1	-0.30 ms	0.08	-3.62	177	**0.006**
F	-1	M	0	1.83 ms	0.08	22.29	173	**< .001**
F	-1	M	1	1.17 ms	0.08	14.28	174	**< .001**
F	0	F	1	-0.63 ms	0.07	-8.48	352	**< .001**
F	0	M	0	-0.001 ms	0.08	-0.02	182	1.000
F	0	M	1	-0.66 ms	0.08	-8.26	183	**< .001**
F	1	M	1	-0.04 ms	0.08	-0.47	183	1.000
M	-1	F	0	2.13 ms	0.08	26.48	183	**< .001**
M	-1	F	1	1.50 ms	0.08	18.69	183	**< .001**
M	-1	M	0	2.13 ms	0.08	25.74	353	**< .001**
M	-1	M	1	1.47 ms	0.08	17.74	352	**< .001**
M	0	F	1	-0.63 ms	0.08	-7.77	183	**< .001**
M	0	M	1	-0.66 ms	0.08	-8.02	352	**< .001**

Table S3.4: Simple effects of relevant predictors exerting a significant effect on z-scored RTs. (A) Simple effect omnibus tests of Sex on Health Condition. **(B)** Simple effect parameter estimates of Sex on Health Condition. **(C)** Simple effect omnibus tests of Flanker Congruency on Sex. **(D)** Simple effect parameter estimates of Flanker Congruency on Sex. A p-value (*p*) < 0.05 was considered statistically significant and highlighted in bold. Df: degrees of freedom; Num df: numerator df; Den df: denominator df. CI: Confidence Interval; SE: Standard Error; MCI: patients with Mild Cognitive Impairment; HOCs: Healthy Older Controls. M: males; F: females; -1: incongruent trials; 1: congruent trials; 0: neutral trials.

(A)

**Moderator levels**				
**Sex**	**F**	**Num df**	**Den df**	***p***
F	9.55	1.00	27.6	**0.005**
M	0.002	1.00	25.5	0.966

(B)

**Moderator levels**				**95% CI**				
**Sex**	**Contrast**	**Estimate**	**SE**	**Lower**	**Upper**	**df**	**t**	***p***
F	MCI – HOCs	-0.22 ms	0.07	-0.37	-0.07	27.6	-3.09	**0.005**
M	MCI – HOCs	-0.003 ms	0.07	-0.16	0.15	25.5	-0.04	0.966

(C)

**Moderator levels**				
**Flanker Congruency**	**F**	**Num df**	**Den df**	***p***
-1	13.14	1.00	154	**<.001**
0	0.00	1.00	160	0.986
1	0.22	1.00	160	0.642

(D)

**Moderator levels**				**95% CI**				
**Flanker Congruency**	**Contrast**	**Estimate**	**SE**	**Lower**	**Upper**	**df**	**t**	***P***
-1	M – F	0.30 ms	0.08	0.14	0.46	154	3.63	**<.001**
0	M – F	0.001 ms	0.08	-0.16	0.16	160	0.02	0.986
1	M – F	0.04 ms	0.08	-0.12	0.20	160	0.47	0.642

S4: Full results of the ANOVA examining the Health Condition * Sex interaction effects on attentional network indices.

Table S4.1: Full results of the ANOVA conducted on the attentional network indices, calculated by subtracting the respective proportional scores. An ANOVA was carried out with Health Condition (MCI and HOCs) and Sex (males and females) as fixed effects and the alerting **(A),** orienting **(B)** and conflict **(C)** indices as the dependent variables. A p-value (*p*) < 0.05 Bonferroni corrected was considered statistically significant and highlighted in bold. Num df: numerator df; Den df: denominator df.

(A)

**Fixed effects**	**F**	**Num df**	**Den df**	***p***	η^2
Health Condition	0.05	1	32	0.827	0.001
Sex	2.82	1	32	0.103	0.081
Health Condition * Sex	0.06	1	32	0.802	0.002

(B)

**Fixed effects**	**F**	**Num df**	**Den df**	***p***	η^2
Health Condition	0.27	1	32	0.605	0.008
Sex	0.71	1	32	0.405	0.021
Health Condition * Sex	0.43	1	32	0.518	0.013

(C)

**Fixed effects**	**F**	**Num df**	**Den df**	***p***	η^2
Health Condition	1.82	1	32	0.187	0.045
Sex	4.66	1	32	**0.039**	0.116
Health Condition * Sex	1.82	1	32	0.187	0.045

Table S4.2: Post Hoc comparisons of Sex showing a significant effect on the conflict index. A p-value (*p*) < 0.05 Bonferroni corrected was considered statistically significant. Δ: estimated marginal means difference; SE: Standard Error; df: degrees of freedom; M: males; F: females.

**Comparison**							
**Sex**	**Sex**	**Δ**	**SE**	**df**	**t**	***p bonf***	**Cohen's *d***
F	M	-0.05	0.02	32.0	-2.16	**0.039**	-0.724

S5: Full results of repeated-measures analysis of variance (ANOVA) examining the Health Condition * Sex interaction effects on mean number of errors in congruent and incongruent trials.

Table S5.1: Full results of the ANOVA conducted on the accuracy rates. An 2x2x2 ANOVA was performed with Health Condition (MCI and HOCs) and Sex (males and females) as between-subjects factors and mean number of errors at both congruent and incongruent trials as the two-levels within-subjects factor. A p-value (p) < 0.05 Bonferroni corrected was considered statistically significant and highlighted in bold. Num df: numerator df; Den df: denominator df.

	F	Num df	Den df	p	η^2
**Within-subjects effects**					
Flanker Congruency	8.12	1	32	**0.008**	0.009
Flanker Congruency * Sex	4.22	1	32	**0.048**	0.005
Flanker Congruency * Health Condition	3.14	1	32	0.086	0.004
Flanker Congruency * Sex * Health Condition	4.95	1	32	**0.033**	0.006
**Between-subjects effects**					
Sex	0.88	1	32	0.356	0.008
Health Condition	10.58	1	32	**0.003**	0.091
Sex * Health Condition	1.34	1	32	0.256	0.012

Table S5.2: Post Hoc comparisons of relevant factors exerting a significant effect on accuracy rates (A) Post Hoc comparisons of Flanker Congruency. **(B)** Post Hoc comparisons of Health Condition. **(C)** Post Hoc comparisons of Flanker Congruency * Sex. **(D)** Post Hoc Comparisons of Flanker Congruency * Sex * Health Condition. A p-value (p) < 0.05 Bonferroni corrected was considered statistically significant and highlighted in bold. Δ: estimated marginal means difference; SE: Standard Error; df: degrees of freedom; HC: Health Condition; MCI: patients with Mild Cognitive Impairment; HOCs: Healthy Older Controls. M: males; F: females. Congruency: Flanker Congruency; -1: incongruent trials; 1: congruent trials; 0: neutral trials.

(A)

Comparison						
**Congruency**	**Congruency**	**Δ**	**SE**	**df**	**t**	***p bonf***
1	-1	-3.14	1.10	32.0	-2.85	**0.008**

(B)

Comparison						
**HC**	**HC**	**Δ**	**SE**	**df**	**t**	***p bonf***
HOCs	MCI	-9.89	3.04	32.0	-3.25	**0.003**

(C)

Comparison								
**Congruency**	**Sex**	**Congruency**	**Sex**	**Δ**	**SE**	**df**	**t**	***p bonf***
1	F	1	M	0.59	2.61	32.0	0.22	1.000
		-1	F	-5.40	1.47	32.0	-3.68	**0.005**
		-1	M	-0.29	3.30	32.0	-0.09	1.000
	M	-1	F	-5.99	3.17	32.0	-1.89	0.408
		-1	M	-0.88	1.64	32.0	-0.53	1.000
-1	F	-1	M	5.11	3.75	32.0	1.36	1.000

(D)

Comparison										
**Congruency**	**Sex**	**HC**	**Congruency**	**Sex**	**HC**	**Δ**	**SE**	**df**	**t**	***p bonf***
1	F	HOCs	1	F	MCI	-9.00	3.48	32	-2.58	0.407
			1	M	HOCs	-0.48	3.69	32	-0.13	1.000
			1	M	MCI	-7.35	3.69	32	-1.99	1.000
			-1	F	HOCs	-1.00	2.08	32	-0.48	1.000
			-1	F	MCI	-18.80	4.31	32	-4.36	**0.004**
			-1	M	HOCs	-1.85	4.66	32	-0.40	1.000
			-1	M	MCI	-7.73	4.66	32	-1.66	1.000
		MCI	1	M	HOCs	8.53	3.69	32	2.31	0.774
			1	M	MCI	1.65	3.69	32	0.45	1.000
			-1	F	HOCs	8.00	4.31	32	1.86	1.000
			-1	F	MCI	-9.80	2.08	32	-4.72	**0.001**
			-1	M	HOCs	7.15	4.66	32	1.53	1.000
			-1	M	MCI	1.28	4.66	32	0.27	1.000
	M	HOCs	1	M	MCI	-6.88	3.89	32	-1.77	1.000
			-1	F	HOCs	-0.53	4.48	32	-0.12	1.000
			-1	F	MCI	-18.32	4.48	32	-4.09	**0.008**
			-1	M	HOCs	-1.38	2.32	32	-0.59	1.000
			-1	M	MCI	-7.25	4.82	32	-1.50	1.000
		MCI	-1	F	HOCs	6.35	4.48	32	1.42	1.000
			-1	F	MCI	-11.45	4.48	32	-2.55	0.437
			-1	M	HOCs	5.50	4.82	32	1.14	1.000
			-1	M	MCI	-0.38	2.32	32	-0.16	1.000
-1	F	HOCs	-1	F	MCI	-17.80	5.00	32	-3.56	**0.033**
			-1	M	HOCs	-0.85	5.31	32	-0.16	1.000
			-1	M	MCI	-6.73	5.31	32	-1.27	1.000
		MCI	-1	M	HOCs	16.95	5.31	32	3.19	0.088
			-1	M	MCI	11.08	5.31	32	2.09	1.000
	M	HOCs	-1	M	MCI	-5.88	5.59	32	-1.05	1.000
